# Variable Craniofacial Shape and Development among Multiple Cave-Adapted Populations of *Astyanax mexicanus*

**DOI:** 10.1093/iob/obae030

**Published:** 2024-08-14

**Authors:** N Holtz, R C Albertson

**Affiliations:** Graduate Program in Organismic and Evolutionary Biology, University of Massachusetts, Amherst, MA 01003, USA; Department of Biology, University of Massachusetts, Amherst, MA 01003, USA

## Abstract

*Astyanax mexicanus* is a freshwater fish species with blind cave morphs and sighted surface morphs. Like other troglodytic species, independently evolved cave-dwelling *A. mexicanus* populations share several stereotypic phenotypes, including the expansion of certain sensory systems, as well as the loss of eyes and pigmentation. Here, we assess the extent to which there is also parallelism in craniofacial development across cave populations. Since multiple forces may be acting upon variation in the *A. mexicanus* system, including phylogenetic history, selection, and developmental constraint, several outcomes are possible. For example, eye regression may have triggered a conserved series of compensatory developmental events, in which case we would expect to observe highly similar craniofacial phenotypes across cave populations. Selection for cave-specific foraging may also lead to the evolution of a conserved craniofacial phenotype, especially in regions of the head directly associated with feeding. Alternatively, in the absence of a common axis of selection or strong developmental constraints, craniofacial shape may evolve under neutral processes such as gene flow, drift, and bottlenecking, in which case patterns of variation should reflect the evolutionary history of *A. mexicanus*. Our results found that cave-adapted populations do share certain anatomical features; however, they generally did not support the hypothesis of a conserved craniofacial phenotype across caves, as nearly every pairwise comparison was statistically significant, with greater effect sizes noted between more distantly related cave populations with little gene flow. A similar pattern was observed for developmental trajectories. We also found that morphological disparity was lower among all three cave populations versus surface fish, suggesting eye loss is not associated with increased variation, which would be consistent with a release of developmental constraint. Instead, this pattern reflects the relatively low genetic diversity within cave populations. Finally, magnitudes of craniofacial integration were found to be similar among all groups, meaning that coordinated development among anatomical units is robust to eye loss in *A. mexicanus*. We conclude that, in contrast to many conserved phenotypes across cave populations, global craniofacial shape is more variable, and patterns of shape variation are more in line with population structure than developmental architecture or selection.

## Synopsis (Spanish)

*Astyanax mexicanus* es una especie de pez de agua dulce con morfologías de cueva ciega y morfologías de superficie videntes. Al igual que otras especies trogloditas, las poblaciones de *A. mexicanus* que habitan en cavernas evolucionaron independientemente y comparten varios fenotipos estereotipados. Entre ellos, la clave es la expansión de ciertos sistemas sensoriales y la pérdida de pigmentación y ojos. Aquí, evaluaremos hasta qué punto existen ciertos paralelismos en el desarrollo craneofacial entre poblaciones de cavernas conevolucion independientemente. Es posible que múltiples factores estén presentes actuando sobre la variación en el sistema de *A. mexicanus*, lo que lleva a varios posibles resultados. Por ejemplo, la regresión ocular se pudo haber desencadenado con una serie conservada de eventos de desarrollo compensatorio, en cuyo caso esperaríamos observar fenotipos craneofaciales muy similares en todas las poblaciones de cavernas. Además, dadas las demandas metabólicas y estructurales del ojo, su ausencia puede constituir una liberación de restricciones, lo que lleva a una expansión de la variaciónes craneofacial, la observación de diferentes patrones de covariación en relación con los peces de superficie, o ambos. Alternativamente, la selección para buscar alimento en cuevas específicas junto con la pérdida de ojos pudo haber desencadenado una línea evolutiva hacia un fenotipo craneofacial conservado. La variación intra e interpoblacional refleja en la historia evolutiva de *A. mexicanus* y en fuerzas demográficas como el flujo, la deriva y los cuellos de botella de genes. Nuestros resultados no respaldan un fenotipo craneofacial conservado en todas las cuevas, ya que casi todas las comparaciones por pares fueron estadísticamente significativas. Sin embargo, las poblaciones adaptadas a cuevas comparten ciertas características anatómicas. También observamos que la disparidad morfológica fue menor entre las tres poblaciones de cuevas en comparación con los peces de la superficie, lo que sugiere que la pérdida de ojos no está asociada con una mayor variación. En cambio, este patrón refleja la diversidad genética relativamente baja en las cuevas en comparación con las poblaciones de la superficie. Las magnitudes de la integración craneofacial también fueron similares entre todos los grupos, lo que significa que el desarrollo coordinado entre unidades anatómicas es resistente a la pérdida ocular en *A. mexicanus*. Concluimos que, a diferencia de muchos fenotipos conservados en las poblaciones de cuevas, la forma craneofacial global es más variable y los patrones de variación de la forma están más en línea con la estructura de la población que con el desarrollo arquitectural.

## Introduction

Naturalists have had a concept of morphological variation since Aristotle wrote *De generatione animalium* in the third-century BC (Aristotle 1910), and the idea of the differential survival of variants has been around since at least al-Jāhiz's, *Kitāb al-Ḥayawān*, in the ninth-century AD (Kopf 1953). Generations of conceptual and technological advancements have refined our ability to quantify, model, and explain the underlying origins of variation, which remain foundational concepts across biological disciplines (Hallgrímsson and Hall 2005). Establishing and maintaining biodiversity depends on the manifestation of variation within diverse genetic, developmental, and ecological systems (Hall 2003; Gilbert et al. 2015). Paradoxically, these same systems also act to limit biodiversity, as each provides constraints on what variation is possible, leaving certain areas of phenotypic space conspicuously empty (Lauder 1981a; Sherratt et al. 2017; Kempes et al. 2019; Bizzarri et al. 2020). Thus, the flip side of diversity is constraint.

Constraints often arise via tradeoffs, whereby expanded expression of one trait results in the muted expression of another trait, and may originate at one of several different biological levels. For example, genetic tradeoffs include negative pleiotropy (Liberles et al. 2011; Percival et al. 2018). Developmental tradeoffs might relate to a system defined by a limited number of stem cell progenitors, such that expanded differentiation of one cell type will directly or indirectly reduce one or more other cell types (Brandon et al. 2023). In biomechanical systems that often characterize organismal architecture, tradeoffs exist between speed and force, such that modular systems may optimize producing speed or power (Wainwright 2007; Holzman et al. 2008; Conith and Albertson 2021). Breaking constraints, or bypassing tradeoffs, can result in dramatic shifts in phenotype and are often associated with expanded ecological or evolutionary success, or both (Cooper et al. 2010; Nagashima et al. 2012; Conith et al. 2018; Gilbert et al. 2020; Sadier et al. 2021; Gilbert et al. 2022). Variation and variability can also be limited or shaped by evolutionary history and population structure. Specifically, population genetic theory predicts that forces such as migration/gene flow, drift, and bottlenecking can dramatically alter patterns and magnitudes of phenotypic variation, acting to release, constrain, or bias phenotypic evolution (Harris 2010; Keller et al. 2013; Dlugosch et al. 2015; Manthey et al. 2020; Fang et al. 2021). Understanding the forces that influence patterns and magnitudes of variation can shed light on evolutionary mechanisms, patterns, and processes.

*Astyanax mexicanus* provides a unique and powerful system to study phenotypic evolution owing to its extreme adaptations and complex evolutionary history. Thirty-four independently evolved *A. mexicanus* cave populations exist alongside river-dwelling surface morphs (Miranda-Gamboa et al. 2023). At least two genetically distinct surface lineages gave rise to different cave populations, and forces such as gene flow, drift, and bottlenecks further shape the current population structure (Gross 2012; Bradic et al. 2013). Nevertheless, cave populations have independently and repeatedly evolved eye loss, albinism, and expanded sensory and foraging structural traits, as well as altered behaviors compared to their surface counterparts (reviewed by Jeffery 2020). There have been many investigations into the genetic and developmental mechanisms underlying the evolution of cave-adapted traits in this system (reviewed by Rohner 2023). For instance, eye development initiates in cavefish but arrests soon after starting (Jeffery 2009). The retention of vestigial eyes in nearly every cave species examined is likely due to the linkage between early eye formation and forebrain patterning (Borowsky 2018). Investigations into the developmental origins of eye loss in *A. mexicanus* have provided insights into how seemingly independent traits influence one another over development. For instance, lens ablation experiments on surface morphs during early embryogenesis resulted in morphological changes within the orbital bones that mimicked cavefish bones (Yamamoto et al. 2003; Dufton et al. 2012; Dufton and Franz-Odendaal 2015). Furthermore, genetic manipulation of eye development in this species showed unexpected effects on jaw/mouth width, an expanded trait in cave morphs, providing evidence for negative pleiotropy in the system (Yamamoto et al. 2009). These studies point to surprising linkages between traits and hint at developmental tradeoffs (Franz-Odendaal and Hall 2006), leading to the question of whether eye loss constitutes the release of a constraint—genetic, developmental, metabolic, or otherwise—and, if so, what the consequences are for the craniofacial skeleton. Given the constellation of extreme adaptations in *A. mexicanus*, combined with their unique demographics, this system offers a robust opportunity to explore, and attempt to disentangle, the forces shaping phenotypic variation.

Here, we quantified and compared craniofacial variation over ontogeny in surface and multiple cave-dwelling *A. mexicanus* populations. We endeavored to capture global aspects of craniofacial anatomy, but focused on regions of the head associated with feeding kinematics, including the lower jaw/mandible, hyoid, and craniofacial profile (Cooper et al. 2011; Ferry et al. 2015; Wainwright et al. 2015). This work is motivated by three, nonmutually exclusive, hypotheses with respect to forces that shape the craniofacial skeleton, including selection, constraint, and biogeography. Adaptation to a cave environment is associated with highly conserved—often stereotypic—changes across multiple phenotypes. Thus, due to strong selection in these extreme environments, we hypothesize that there is a conserved “cave” craniofacial phenotype across populations. In addition, if the eye imposes a constraint on other developmental systems (e.g., skeletal; Franz-Odendaal and Hall 2006), then we hypothesize that removing this constraint will lead to increased disparity, and/or changes in the covariance structure, of the craniofacial skeleton in cavefish versus surface fish. An expectation of both the selection and constraint hypothesis is that cave phenotypes will be similar and equally divergent from surface phenotypes. An alternate hypothesis is that patterns and magnitudes of phenotypic variation will align with *A. mexicanus* population structure, which has been well characterized via genome-wide genotypic data (e.g., Bradic et al. 2013). Collectively, these data will provide insights into the forces influencing craniofacial variation across populations of this unique species. We discuss our results in the context of evo-devo, demographics, and the functional morphology of feeding.

## Methods

### Specimen processing and digitization

All specimens were lab-raised individuals from populations originating in Mexico's Sierra de El Abra region. Cave morph lines originate from Molino, Tinaja, and Pachon caves. Surface morph lines originated from the Río Choy river of Sierra de El Abra. Cave morphs represent just 3 of 34 known caves populated by cave-adapted *A. mexicanus* (Miranda-Gamboa et al. 2023). While this is only a subset, these caves are well documented in the literature, are of different ages, and represent multiple *A. mexicanus* lineages (Gross 2012, but also see Fumey et al. 2018), allowing us to compare morphological trends across multiple evolutionary events.

Populations were sampled at three life history stages. Stage 1 represented 4- to 7-day larvae. Stage 2 were 30- to 60-day juveniles. Stage 3 were 90+-day subadults. These stages were chosen to capture shape before and after dietary niche shifts that occur in nature (i.e., between stages 2 and 3; Espinasa et al. 2017). Sizes and sample sizes are provided in Table 1. At each stage, samples were fixed, cleared, and stained to preserve and visualize cartilaginous and bony structures. A 4% solution of paraformaldehyde (Sigma-Aldrich, Co., St. Louis, MO USA) in 1% phosphate buffered saline (PBS) (Fisher Scientific, Fair Lawn, NJ USA) was used for fixation. Before fixation, specimens were first anesthetized using a low dose of MS222 (Syndel, Ferndale, WA USA), and then euthanized with a combination of ice water and high dose of MS222 following protocols approved by the UMass IACUC. This procedure allowed the craniofacial skeleton to be fixed in a natural “resting” state. Following a 1+-day fixation, specimens were cleared using trypsin (Electron Microscope Sciences, Hatfield, PA USA) and double-stained using acid-free Alcian (Sigma-Aldrich, Co., St. Louis, MO USA) blue for cartilage and Alizarin (Sigma-Aldrich, Co., St. Louis, MO USA) red for bony elements (Walker and Kimmel 2007). Prior to imaging, specimens were stored in 100% glycerol (Fisher Scientific, Fair Lawn, NJ USA) with thymol (Consolidated Chemical and Solvenix LLC, Quakertown, PA USA) crystals as an antifungal.

**Table 1:** Size ranges, as total head length, reported in millimeter, and head length measured as the distance between the back of the skull (where the spine/notochord inserts) and the rostral tip of the upper jaw^a^

	**St1**	**St2**	**St3**
Head length (mm)			
Molino	0.71–1.05	1.57–2.01	4.75–5.14
Pachon	0.83–0.99	1.43–1.93	4.45–5.09
Surface	0.88–1.11	1.38–2.34	4.12–5.12
Tinaja	0.89–1.06	1.42–2.35	4.55–5.43
Sample sizes			
Molino	13	28	7
Pachon	14	17	8
Surface	12	21	6
Tinaja	12	21	8

^a^Sample sizes across populations: stages are also reported.

**Fig. 1:**
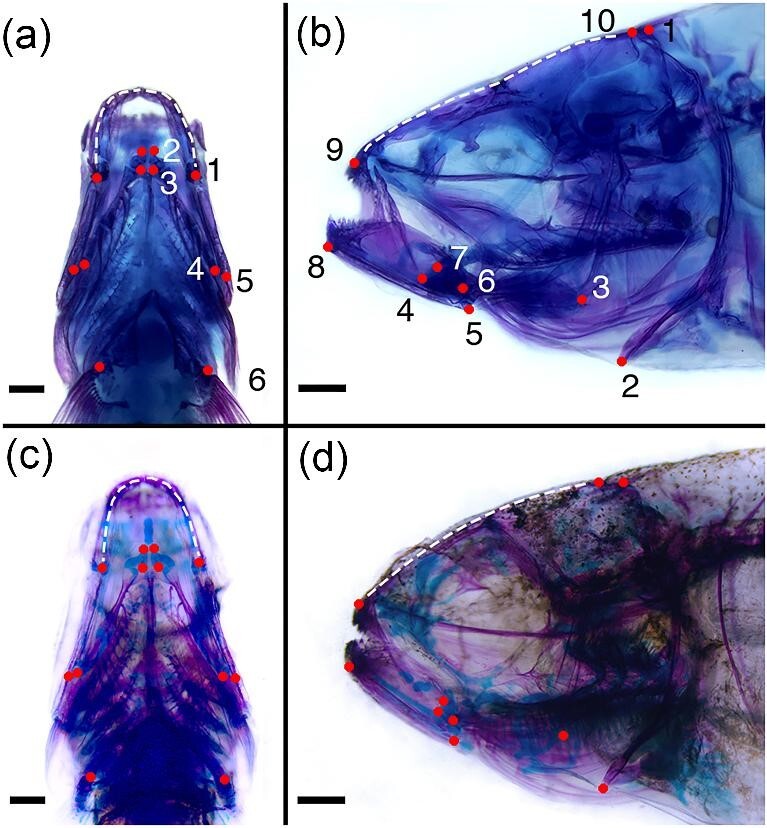
Stage 3 cleared and double-stained *Astyanax* specimens are shown. Landmark (LM) schemes are illustrated on the Pachon individual at top (**a, b**), and surface fish at bottom (**c, d**). Landmarks are denoted as solid red dots, while curves along which semi-landmarks were placed are denoted as dashed white lines. In the ventral view, LM 1 depicts the mandibular–quadrate joint, LMs 2 and 3 define the anterior end of the hyoid, LM 4 is the hyoid–interhyal joint, LM 5 is the interhyal–hyomandibula joint, and LM 6 is the proximal end of the ventral most pectoral fin ray/pad (stage 1). Contralateral LMs were used to capture bilateral shape variation. In addition, we used nine semi-landmarks between the left and right mandibular LMs. In the lateral view, LMs 1 and 2 depict the dorsal and ventral-most ends of the cleithrum, LMs 3 and 4 define the posterior and anterior-most ends of the hyoid, LM 5 is the ventral tip of the retroarticular process, LM 6 is the mandibular–quadrate joint, LM 7 is where the coronoid meets the articular bone, LM 8 is the ventral edge of the distal end of the mandible, LM 9 is the tip of the premaxilla/upper jaw (stage 1), and LM 10 is the posterior end of the parietal bone (in stage 1 and 2 animals this landmark was placed at the posterior end of the dorsal cranium). In this view, we also used three semi-landmarks between LMs 9 and 10 to assess variation in cranial profile. Scale bars equal 500 μm in all panels.

Each animal was photographed in the lateral and ventral view using a Leica DFC450 C digital camera under a Leica M165 FC stereo microscope. Insect pins and modeling clay were used to orient animals for photography. Specimens were imported into StereoMorph (Olsen and Westneat 2015) and landmarked by a single investigator (N.H.). Ventral landmarks (and semi-landmarks) were chosen to cover the anterior-most point of the mandibular symphysis to the beginning of the pectoral fin rays (Fig. 1a, c). Similarly, lateral landmarks (and semi-landmarks) were chosen to capture geometry from the distal tips of the upper and lower jaw to the cleithrum (pectoral girdle; Fig. 1b, d). Since shape variation associated with the orbit is well described in this species (e.g., Dufton et al. 2012; Dufton and Franz-Odendaal 2015; Gross et al. 2014; Powers et al. 2017), we intentionally did not include landmarks in this region, and instead focused on the mandible, hyoid, and craniofacial profile, which are linked to fish feeding performance (Cooper et al. 2011; Ferry et al. 2015; Wainwright et al. 2015). For instance, jaw width is associated with mouth gape, hyoid length and position can affect the dynamics of hyoid depression, and craniofacial profile can influence the direction of jaw protrusion. Variation in these traits is associated with variation in feeding mode across life history stages and among fish species (Lauder 1980; Hernández et al. 2002; Van Wassenbergh et al. 2006; Cooper et al. 2010; Powder et al. 2015). Landmarks and semi-landmarks are described in the legend of Fig. 1.

### Geometric morphometric analyses

All statistics were performed on ventral and lateral datasets and conducted in Geomorph (Adams et al. 2018). Because forces such as selection and constraint may have different effects at different life history stages, we examined shape variation at multiple timepoints over ontogeny. Landmark coordinates were first aligned via generalized Procrustes analysis (Goodall 1991) using the *gpagen* function. Procrustes ANOVA was then run following (Gilbert et al. 2020) across cave locality within each stage. The analyses involved comparing the null model (shape ∼ size) to the full model (shape ∼ size + population). Centroid size (CS) was used as our size metric. In this way, we evaluated the effects of locality on shape, accounting for the effect of size. We next quantified differences in developmental trajectories between populations using the *trajectory.analysis* function, which uses ANOVA (e.g., shape ∼ population * stage) to compare differences in trajectory length, angle, and shape (Collyer and Adams 2013). Data were visualized using principal component analyses (PCA) alongside associated deformation grids. Convex hulls superimposed over PCA plots delineated the “shape space” occupied by each group, allowing the visualization of morphological variation in two dimensions (principal component—PC1 and PC2). Procrustes variances were then obtained and compared between populations within each stage, as well as across stages, using the *morphol.disparity* function. Finally, we used the *two.b.pls* function to run a two-block partial least squares (PLS) test to assess degrees of correlation between various landmark configurations, including between lateral and ventral datasets, as well as within ventral and lateral landmark datasets. In the ventral view we compared the mandible versus hyoid landmark blocks. Laterally, we compared the pharyngeal skeletal versus cranial profile landmark blocks. All statistical analyses used a randomized residual permutation procedure (RRPP), which subjected landmark data to 10,000 random permutations (Collyer and Adams 2018).

## Results

### Craniofacial shape differs across populations at each developmental stage

Procrustes ANOVA revealed significant differences in mean shape for nearly all pairwise comparisons in the ventral and lateral (Table 2) views. Only three comparisons were insignificant at the 0.05 level, of which all were between the Pachon and Tinaja populations. All pairwise surface-to-cave comparisons were significant and generally involved the most robust effect sizes (e.g., *Z* scores).

**Table 2:** Output of Procrustes ANOVA, showing both the *Z* score and *P* values for each pairwise comparison^a^

	**Ventral view**		**Lateral view**	
**St1**	***Z* score**	***P* value**	***Z* score**	***P* value**
Molino:Pachon	***1.829***	***0.0334***	***4.241***	***0.0001***
Molino:surface	***3.332***	***0.0001***	***4.294***	***0.0001***
Molino:Tinaja	***1.652***	***0.0511***	***4.474***	***0.0001***
Pachon:surface	***3.145***	***0.0004***	***4.319***	***0.0001***
Pachon:Tinaja	0.618	0.2721	***3.751***	***0.0002***
Surface:Tinaja	***3.871***	***0.0001***	***3.864***	***0.0001***
**St2**				
Molino:Pachon	***2.777***	***0.0008***	***4.365***	***0.0001***
Molino:surface	***3.299***	***0.0001***	***3.606***	***0.0001***
Molino:Tinaja	***4.293***	***0.0001***	***5.760***	***0.0001***
Pachon:surface	***2.646***	***0.0015***	***2.311***	***0.0096***
Pachon:Tinaja	***2.378***	***0.0061***	***2.165***	***0.0153***
Surface:Tinaja	***3.463***	***0.0001***	***4.276***	***0.0001***
**St3**				
Molino:Pachon	***2.419***	***0.0057***	***2.003***	***0.0245***
Molino:surface	***2.749***	***0.0017***	***3.417***	***0.0003***
Molino:Tinaja	***1.793***	***0.0378***	***1.867***	***0.0323***
Pachon:surface	***3.341***	***0.0002***	***3.331***	***0.0002***
Pachon:Tinaja	1.136	0.1360	0.148	0.4344
Surface:Tinaja	***3.998***	***0.0001***	***4.243***	***0.0001***

^a^Values are bold-faced and italicized when *P* < 0.05.

**Fig. 2:**
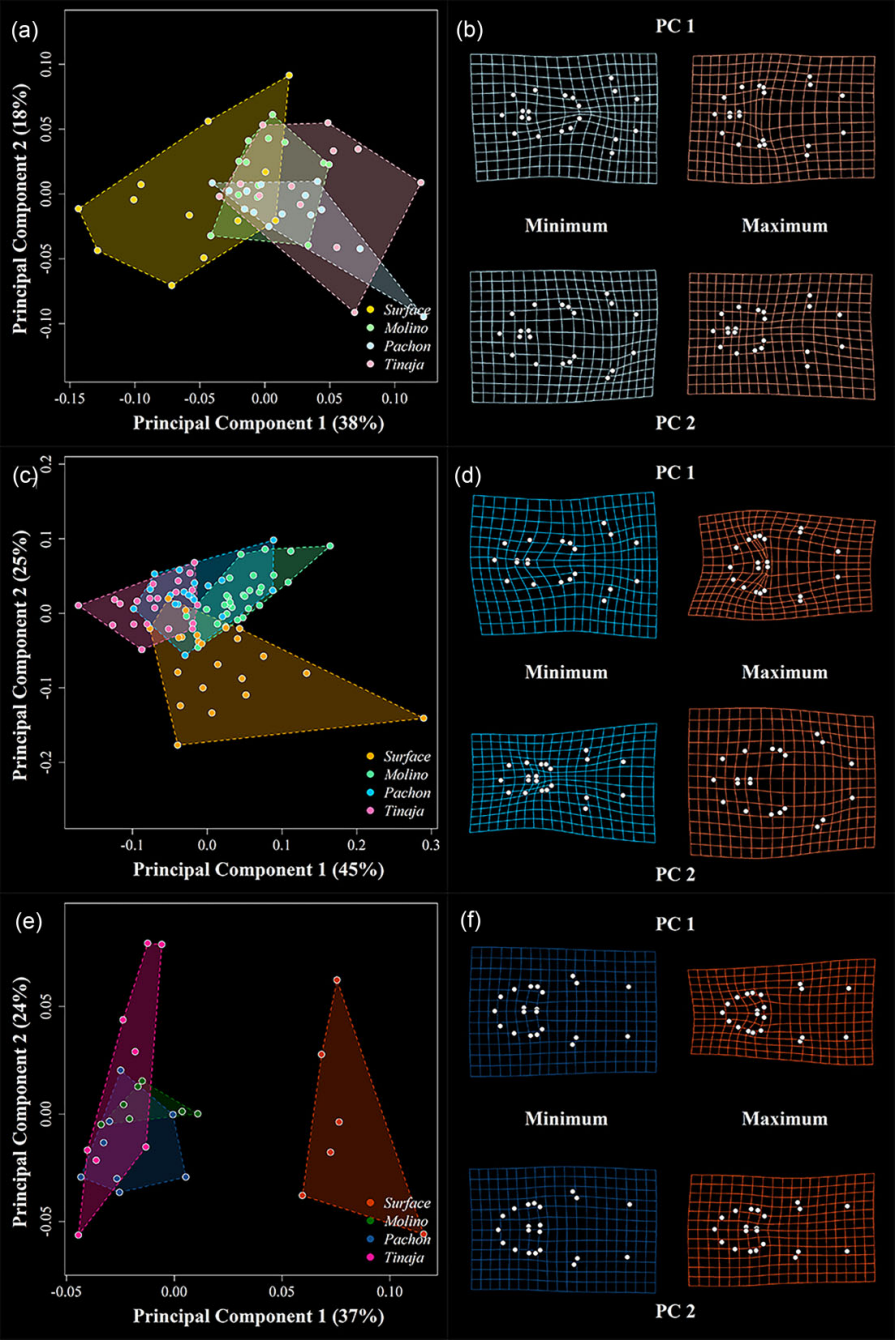
Principal component analysis (PCA) and associated deformation grids for the ventral landmark dataset. Data for stage 1 (**a, b**), stage 2 (**c, d**), and stage 3 (**e, f**) are shown separately. For each stage, the percent variation explained by each axis is provided, and the shape space is overlain by colored convex hulls for each locality. Deformation grids represent the predicted shape at minimum and maximum values along each axis as compared to mean shape. Anterior is to the left, posterior is to the right.

We next used PCA to visualize patterns of morphological variation at each stage. In the ventral view (Fig. 2), surface morphs occupy a distinct area of morphospace compared to cave morphs at every stage and exhibit a nonoverlapping distribution at the adult stage (Fig. 2a, c, e). Deformation grids capture variation in shape that largely explains the relative width/length of the mandible and hyoid, especially at stages 1 and 2 (Fig. 2b, d, f). Surface fish segregate from cave populations along PC1 at stage 1, and possess thinner heads in comparison (Fig. 2a, b). At stage 2, surface fish are distinct from cavefish along PC2, and possess relatively thin mandibles and hyoid bones compared to cavefish (Fig. 2c, d). We note that variation in cavefish populations is relatively constrained along PC2, while surface fish cover a more substantial range of PC2 morphospace. At stage 3, variation along both PC1 and PC2 captures differences in mandible width, shape, and the relative width of the posterior head. Surface fish are distinct from cavefish along PC1 and have—on average—narrow, V-shaped mandibles compared to cavefish, which possess wider U-shaped mandibles (Fig. 2f). Both surface and Tinaja populations exhibit high variation along PC2, while Molino and Pachon localities exhibit more constrained variation along this axis.

**Fig. 3:**
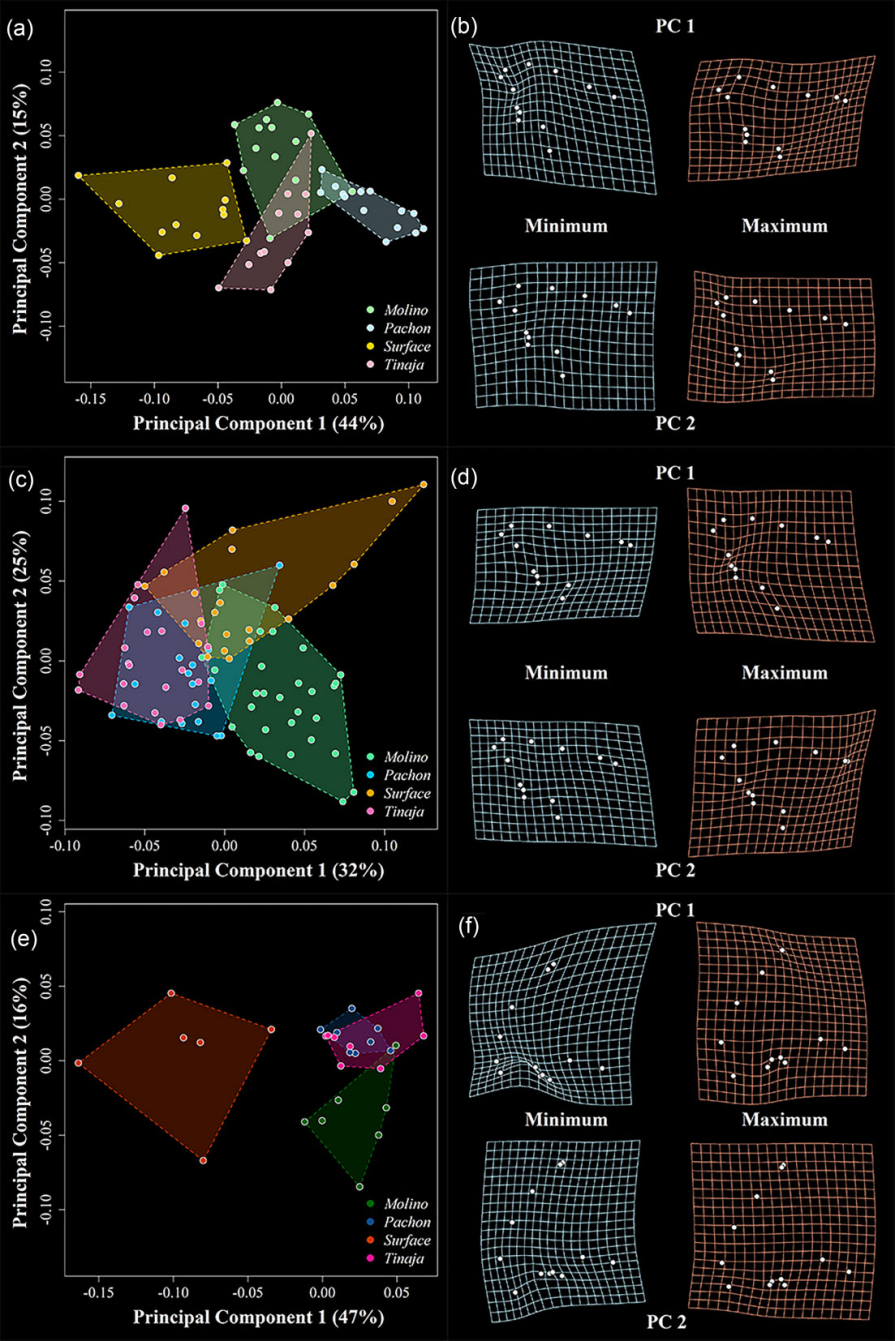
Principal component analysis (PCA) and associated deformation grids for the lateral landmark dataset. Data for stage 1 (**a, b**), stage 2 (**c, d**), and stage 3 (**e, f**) are presented separately. For each stage, the percent variation explained by each axis is provided, and shape space is overlain by colored convex hulls for each locality. Deformation grids represent the predicted shape at minimum and maximum values along each axis as compared to mean shape. In **b** and **d**, anterior is facing the top left corner, while posterior is bottom right. In **f**, anterior is bottom left, while posterior is top right.

PC morphospace for the lateral perspective reveals similar trends insofar as surface fish diverging along PC1 at stages 1 and 3 and along PC2 at stage 2 (Fig. 3). At stage 1, all populations occupy relatively distinct regions of morphospace compared to the ventral view. Variation along PC1 describes variation in head profile and the anterior–posterior positioning of the ceratohyal bone, with cavefish possessing relatively concave skull profiles and anteriorly shifted ceratohyal bones. PC2 also captures aspects of variation in skull profile and head depth at this stage and mainly separates Tinaja and Molino cave populations (Fig. 3a, b). At stage 2, unlike the ventral shape data, cavefish populations exhibit a high degree of variation along both axes, and both PC1 and PC2 described variation in craniofacial profile and positioning of the ceratohyal bone. At this stage, the more rounded profiles of surface fish develop while they retain posteriorly shifted ceratohyal bones (Fig. 3c, d). The most robust differences between cave and surface morphs are at stage 3, where—similar to the ventral view—surface and cave populations exhibit nonoverlapping distributions in PC space (Fig. 3e, f). Deformation grids show that surface fish possess more rounded preorbital profiles and upturned jaws than cavefish.

While PC1 appears to be introducing artifacts associated with variation in jaw opening, we assert that this aspect of variation is biologically rooted, and may represent a previously unnoted aspect of cavefish anatomy. Mainly, we found that all cavefish samples had jaws that were slightly agape, whereas surface fish did not. Since all animals were prepared for analysis in the same way, it is unlikely that fixation artifact only affected cavefish samples. Rather, what seems to be the case is that the physiological resting state in cavefish is associated with jaws that are slightly agape (Fig. 1). We expand on this idea in the Discussion section. Further, the mandible is deeper in cavefish compared to surface fish, and the landmark used to capture the distal extent of the lower jaw was placed along the ventral edge of the dentary. Thus, this aspect of variation is accentuated by dentary depth.

### Ontogenetic trajectories differ across populations

**Fig. 4:**
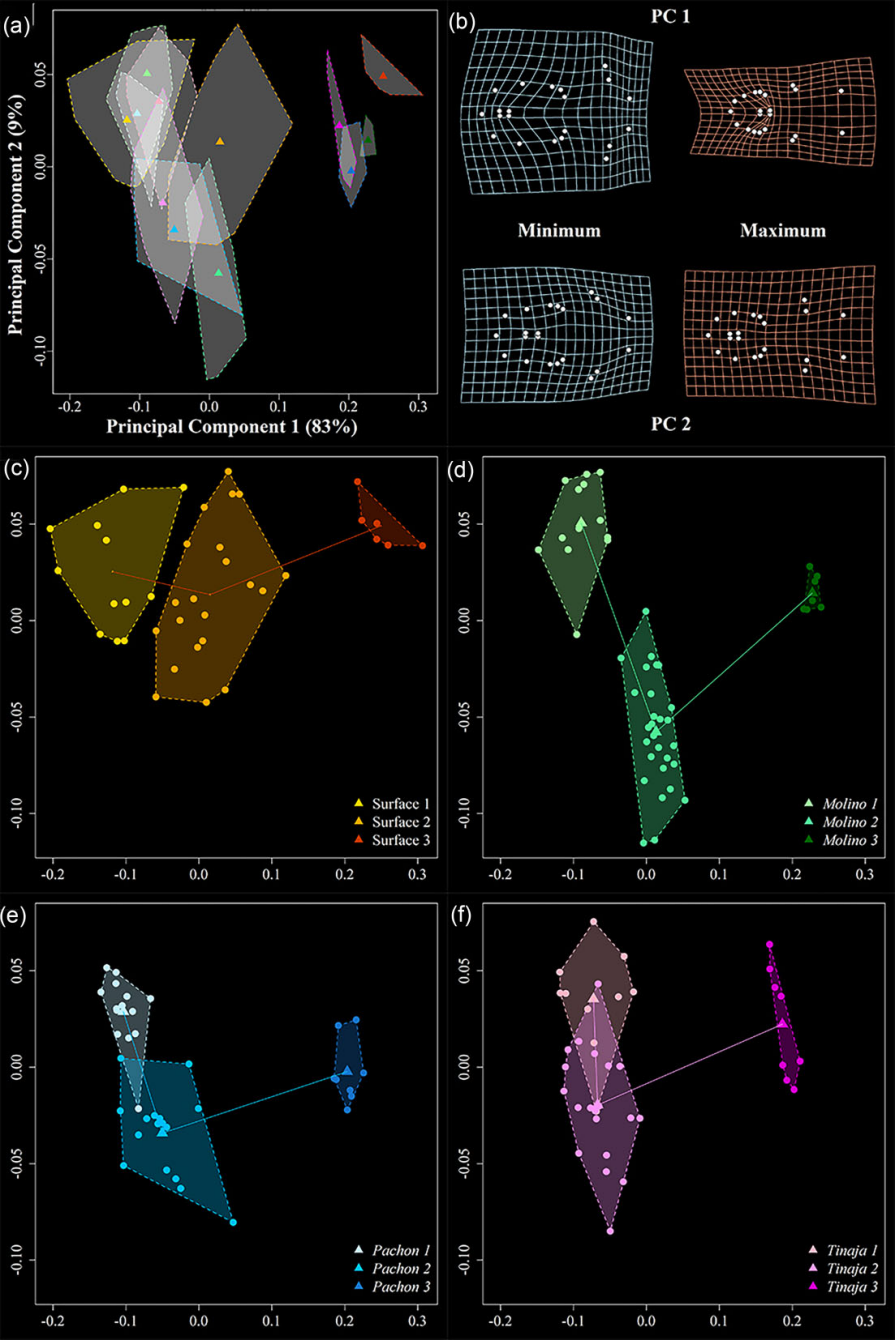
Principal component analysis (PCA) and associated deformation grids for the trajectory analysis in the ventral view. All population: stages are depicted in (**a**), and the percent variation explained by each component is provided. Corresponding deformation grids are illustrated in (**b**) as minimum and maximum values relative to mean shape. The anterior–posterior axis runs left to right. For ease of interpretation, each population is also shown separately (**c–f**). Circles represent individual data points, while triangles represent group means. Populations are denoted by different colors, and increasingly older fish are represented by darker hues.

**Fig. 5:**
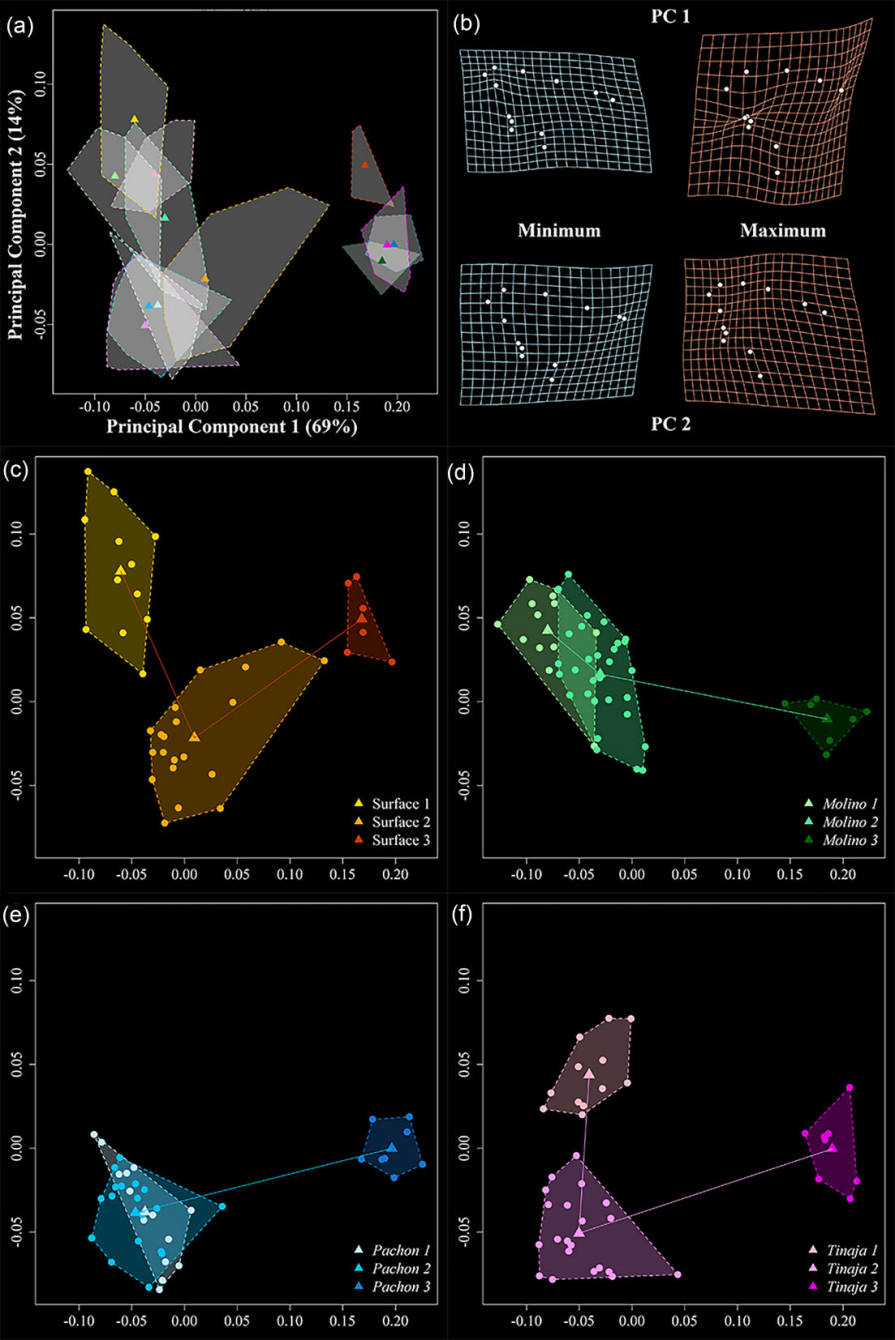
Principal component analysis (PCA) and associated deformation grids for the trajectory analysis in the lateral view. All population: stages are depicted in (**a**), and the percent variation explained by each axis is provided. Corresponding deformation grids are illustrated in (**b**) as minimum and maximum values relative to mean shape. Here, the anterior–posterior axis runs top left to bottom right. For ease of interpretation, each population is also shown separately (**c–f**). Circles represent individual data points, while triangles are group means. Populations are denoted by different colors, with increasingly older fish represented by darker hues.

We next examined craniofacial development across *A. mexicanus* populations within a common PC shape space (Figs. 4 and 5). In the ventral view (Fig. 4), PC1 and PC2 capture 92% of morphological variation. PC1 (83%) describes differences between early stages and stage 3, capturing variation in relative mandible size and positioning of the pectoral fins, with older animals possessing relatively small jaws and posteriorly shifted pectoral fin landmarks, which likely reflects an expansion of the branchial region of the pharyngeal skeleton. PC2 (9%) captures differences between stages 1 and 2 and reflects variation in head width, with stage 2 animals possessing relatively wider jaws. Plotting each population reveals qualitative differences in developmental trajectories, especially when comparing surface fish to cave populations (Fig. 4c–f). These patterns bear out statistically (Table 3), where trajectory angles are distinct between surface and cave populations and similar among cave populations. Trajectory lengths are statistically indistinguishable between populations, whereas trajectory shapes differ for all pairwise comparisons except between Pachon and Tinaja (*P* = 0.0641).

**Table 3:** Output of trajectory analysis, showing the *Z* score and *P* values for each pairwise comparison^a^

	**Ventral view**		**Lateral view**	
**Length**	***Z* score**	***P* value**	***Z* score**	***P* value**
Molino:Pachon	0.354	0.3876	−1.944	0.9750
Molino:surface	−2.750	0.9974	−1.055	0.8350
Molino:Tinaja	0.040	0.5037	1.456	0.0670
Pachon:surface	−0.525	0.7063	−1.529	0.9310
Pachon:Tinaja	−1.269	0.8802	1.193	0.1210
Surface:Tinaja	0.139	0.4744	1.166	0.1270
**Angle**				
Molino:Pachon	−0.368	0.6394	***2.836***	***0.0010***
Molino:surface	***2.781***	***0.0018***	***3.016***	***0.0020***
Molino:Tinaja	0.777	0.2225	***2.687***	***0.0040***
Pachon:surface	***2.393***	***0.0072***	***2.190***	***0.0200***
Pachon:Tinaja	−0.463	0.6798	0.242	0.4160
Surface:Tinaja	***1.798***	***0.0375***	***1.966***	***0.0220***
**Shape**				
Molino:Pachon	***1.670***	***0.0454***	***2.028***	***0.0200***
Molino:surface	***2.513***	***0.0031***	0.594	0.2800
Molino:Tinaja	***3.080***	***0.0003***	***3.013***	***0.0010***
Pachon:surface	***1.597***	***0.0519***	***1.745***	***0.0380***
Pachon:Tinaja	1.532	0.0641	0.914	0.1880
Surface:Tinaja	***3.609***	***0.0001***	***2.062***	***0.0140***

^a^Values are bold-faced and italicized when *P* < 0.05.

In the lateral view (Fig. 5), PC1 and PC2 capture 83% of the total variation, and—similar to the ventral view—PC1 (69%) separates stage 3 from stages 1 and 2. Notably, PC2 (16%) less reliably captures differences between stages 1 and 2, specifically for Molino and Pachon populations where these stages overlap in two-dimensional shape space. PC1 describes variation in the craniofacial profile and anterior positioning of the ceratohyal bone and mandible length, with older fish possessing rounder heads, posteriorly shifted ceratohyals, and mandibles that are shorter relative to overall head length. PC2 mainly captures variation in the positioning of the ceratohyal and mandible length, with stage 2 fish possessing longer jaws and posteriorly shifted ceratohyals on average, but again only for surface and Tinaja populations as Molino and Pachon fish are similar along PC2 at stages 1 and 2. Similar to the ventral view, lateral shape trajectories are statistically indistinguishable across populations for trajectory length but exhibit more pairwise differences, including between cave populations, for trajectory angle and shape (Table 3). The exception, again, is the comparison between Pachon and Tinaja populations, which is statistically indistinguishable for every trajectory metric.

### Craniofacial variation is more canalized in cavefish versus surface fish and becomes more canalized over ontogeny in all populations

The potential of a trait to vary (e.g., variability) has important evolutionary consequences, and can be influenced by several factors including selection, developmental constraint, and genetic/nucleotide diversity (e.g., Taute et al. 2014; Yanagida et al. 2015; Legrand et al. 2016; Agosto and Auerbach 2022). We therefore examined and compared morphological disparity across populations. We noted reduced variation (i.e., canalization) in our two-dimensional PC morphospace within cavefish versus surface fish populations (Figs. 2 and 3). This trend was statistically supported via the calculation and comparison of Procrustes variances (e.g., disparity) within and across samples (Table 4). Specifically, surface fish exhibited the highest Procrustes variance at every stage and in both views, with 10/18 pairwise comparisons reaching statistical significance at the 0.05 level. If only considering stages 1 and 2, 10/12 pairwise comparisons are statistically significant, likely due to a reduction in variation across all populations at stage 3 (Table 4).

**Table 4:** Disparity analyses^a^

	**Molino**	**Pachon**	**Surface**	**Tinaja**		
**Ventral view**						
St1	0.0031905	0.0042761	0.0089315	0.0063759		
St2	0.0051214	0.0045941	0.0133592	0.0048135		
St3	0.0023857	0.0016629	0.0035311	0.0032945		
	**St1**		**St2**		**St3**	
	**Abs. diff.**	***P* value**	**Abs. diff.**	***P* value**	**Abs. diff.**	***P* value**
Molino:Pachon	0.0010856	0.5480	0.0005273	0.8590	0.0007228	0.4977
Molino:surface	***0.0057411***	***0.0011***	***0.0082378***	***0.0002***	0.0011454	0.3049
Molino:Tinaja	0.0031854	0.0862	0.0003080	0.9211	0.0009088	0.3821
Pachon:surface	***0.0046554***	***0.0076***	***0.0087651***	***0.0017***	0.0018683	0.0783
Pachon:Tinaja	0.0020998	0.2503	0.0002193	0.9388	0.0016316	0.1017
Surface:Tinaja	0.0025557	0.1806	***0.0085458***	***0.0008***	0.0002366	0.8251
	**St1**	**St2**	**St3**			
	0.00481478	0.00532821	0.00244252			
	**Abs. diff.**	***P* value**				
St1:St2	0.0005134	0.4749				
St1:St3	***0.0023723***	***0.0116***				
St2:St3	***0.0028857***	***0.0009***				
**Lateral view**						
	**Molino**	**Pachon**	**Surface**	**Tinaja**		
St1	0.0036743	0.0026162	0.0070824	0.0034635		
St2	0.0038543	0.0038506	0.0062362	0.0035245		
St3	0.0030992	0.0015723	0.0052740	0.0028096		
	**St1**		**St2**		**St3**	
	**Abs. diff.**	***P* value**	**Abs. diff.**	***P* value**	**Abs. diff.**	***P* value**
Molino:Pachon	0.0010581	0.3736	0.0000036	0.9980	0.0015269	0.2730
Molino:surface	***0.0034081***	***0.0025***	***0.0023819***	***0.0470***	0.0021748	0.1415
Molino:Tinaja	0.0002108	0.8659	0.0003298	0.7934	0.0002896	0.8374
Pachon:surface	***0.0044662***	***0.0001***	0.0023855	0.0823	***0.0037016***	***0.0061***
Pachon:Tinaja	0.0008473	0.4876	0.0003262	0.8138	0.0012373	0.3648
Surface:Tinaja	***0.0036189***	***0.0009***	***0.0027117***	***0.0393***	0.0024644	0.0747
	**St1**	**St2**	**St3**			
	0.00451277	0.00419975	0.00264559			
	**Abs. diff.**	***P* value**				
St1:St2	0.0003130	0.6353				
St1:St3	***0.0018672***	***0.0266***				
St2:St3	***0.0015542***	***0.0441***				

^a^Procrustes variances are provided for populations across stages, as well as stages across populations. In addition, absolute differences and *P* values are reported for pairwise comparisons. Values are bold-faced and italicized when *P* < 0.05.

### Landmark datasets covary in both cave and surface populations

Covariation (e.g., integration) among traits can influence the direction and/or efficiency of evolutionary change (reviewed by Armbruster et al. 2014). We therefore performed two-block PLS tests to investigate magnitudes of integration in cave and surface populations, beginning with a comparison between ventral and lateral landmark datasets (Table 5). Previous work has shown that eye loss is associated with conformational changes in lateral skull shape (Yamamoto and Jeffery 2000; Yamamoto et al. 2003; Dufton et al. 2012; Dufton and Franz-Odendaal 2015), so cavefish eye loss may lead to distinct patterns of variation in the lateral versus ventral view. If so, cavefish populations should exhibit lower correlation coefficients than surface fish. Alternatively, higher relative magnitudes of covariation between lateral and ventral shapes may be observed in cavefish due to a linkage between eye loss and expanded jaw width (Yamamoto et al. 2009; Atukorala and Franz-Odendaal 2018). We found significant correlations across all populations, with comparable r-PLS values and *Z* scores. Tinaja cavefish returned the highest r-PLS and *Z* score, while Pachon fish returned the lowest values.

**Table 5:** Integration analyses^a^

	**r-PLS**	***Z* score**	***P* value**
**Ventral:lateral**			
Molino	***0.929***	***4.5146***	***0.001***
Pachon	***0.955***	***4.2417***	***0.001***
Surface	***0.879***	***4.4033***	***0.001***
Tinaja	***0.963***	***5.1357***	***0.001***
	**Integration**		
	**r-PLS**	***Z* score**	***P* value**
**Ventral—mandible: hyoid**			
Molino	***0.961***	***4.9265***	***0.001***
Pachon	***0.941***	***4.9938***	***0.001***
Surface	***0.947***	***3.765***	***0.001***
Tinaja	***0.869***	***4.6438***	***0.001***
	**Integration**		
	**r-PLS**	***Z* score**	***P* value**
**Lateral profile: pharyngeal skeleton**			
Molino	***0.887***	***4.7467***	***0.001***
Pachon	***0.850***	***4.3283***	***0.001***
Surface	***0.802***	***4.4129***	***0.001***
Tinaja	***0.932***	***5.2947***	***0.001***

^a^Results are reported for comparisons between ventral and lateral landmark datasets, as well as for within ventral and lateral landmarks. Values are bold-faced and italicized when *P* < 0.05.

We next investigated covariation within ventral and lateral views, using a two-module hypothesis for each. In the ventral view, we assessed the degree of covariation between hyoid and mandible landmarks, finding significant levels of integration between these shapes across all populations with comparable r-PLS values and effect sizes. In the lateral view, we compared variation among landmarks associated with the craniofacial profile to those denoting the pharyngeal skeleton and, again, saw significant levels of integration across all populations with comparable effect sizes. The outcome of these analyses suggests that cave adaptations have not led to a disintegration of covariation levels or patterns in *A. mexicanus*.

## Discussion

### Shaping craniofacial variation: population structure or developmental constraints?

Systems characterized by trait convergence have increasingly become the focus of evolutionary developmental biologists who seek to understand how, or if, conserved features of gene regulatory networks and/or developmental programs bias evolution (Peter and Davidson 2011; Arendt et al. 2016; Peter and Davidson 2016; Smith et al. 2018; Artur and Kajala 2021). Despite widespread convergence in traits across *A. mexicanus* cavefish populations, craniofacial geometry does not follow this pattern. These results highlight flexibility, as opposed to limitations, in developmental systems that govern craniofacial form.

One hypothesis that motivated this study was that the eye represents a constraining force on the skull, owing to the need to integrate this large organ into its architecture. This is similar to the “interacting module” hypothesis of Franz-Odendaal and Hall (2006), whereby different anatomical and functional units interact over development such that selection on one unit will propagate change in other units. They point to the putative association between eyes and bone formation, as well as that between neuromasts and bone, to predict and explain correlative trait evolution in *Astyanax* (Franz-Odendaal and Hall 2006). Ours is an extension of this hypothesis, with a prediction that eye loss should release the skull from an anatomical constraint, resulting in expanded morphological variation in cave versus surface populations. Notably, our data were unsupportive of this prediction; at all stages and in both views, surface fish exhibited the highest levels of disparity compared to any cavefish population. Instead, our data align with work showing that, relative to surface populations, cavefish have low effective population sizes and reduced genetic diversity (Bradic et al. 2012). We cannot reject the hypothesis that eye loss leads to expanded variation in certain dimensions of shape. Indeed, increased variation is well documented in cavefish in terms of the number and size of infraorbital bones surrounding the orbit (Dufton and Franz-Odendaal 2015; Gross et al. 2016). In other words, a more refined hypothesis may be that the eye constrains local—but not global—craniofacial architecture.

Comparisons of craniofacial shape among cavefish populations also reflect the demographic history and gene flow of *A. mexicanus*. Specifically, Pachon and Tinaja populations consistently exhibited the most similar craniofacial shapes (e.g., lowest *Z* scores) across stages and in both perspectives. The only pairwise comparisons in our dataset that were not significant at the 0.05 level were between these two populations at stages 1 and 3. Pachon and Tinaja cavefish originate from the same ancestral surface population, which is genetically distinct from the population that gave rise to the Molino cavefish (Bradic et al. 2013). Further, there has been more gene flow over time between Pachon and Tinaja populations, compared to between either cave and Molino (Bradic et al. 2013). Thus, the phenotypic similarity between Pachon and Tinaja cavefish is matched by their genomic similarity, whereas Molino's distinct demographic history is matched by divergent craniofacial shape relative to Pachon and Tinaja.

### Functional implications of craniofacial shape variation

While our data suggest flexibility in global craniofacial shape across cavefish populations, there were some general similarities. Three anatomical features in particular were shared among cavefish: (1) wide jaws, (2) a relatively flat head, and (3) size/positioning of the hyoid. Expanded jaw width in cavefish relative to surface fish has been noted previously, with ties to eye loss via antagonistic pleiotropy (Yamamoto et al. 2009; Atukorala and Franz-Odendaal 2018), although recent quantitative genetic studies are unsupportive of this mechanism (Oliva et al. 2022; Powers et al. 2023), suggesting that the relationship between eyes and jaws is more complex. A dorsal–ventral flattening of the cavefish skull may also be linked with eye loss, as there is no longer a need for a rounded skull to accommodate the eye; however, this association is speculative.

Notably, all three traits listed above may be involved in enhanced suction feeding in cavefish. As fry, cavefish consume prey requiring suction feeding, including small pelagic invertebrates (Espinasa et al. 2017). As adults, there seems to be a shift away from foraging on prey in the water column (Espinasa et al. 2017); however, adult cavefish continue to use suction to generate a hydrodynamic velocity field around their heads to detect obstacles (Holzman et al. 2014). Thus, the ability to generate suction may be adaptive at multiple life history stages in cavefish. Common anatomical themes among suction-feeding fishes include wide jaws (i.e., wide mouths to increase gape), long/shallow skulls (associated long/isognathus jaws), and long hyoids (to drive hyoid depression and expansion of the buccal cavity) (Gibb and Ferry-Graham 2005; Westneat 2005; Wainwright et al. 2007; Holzman et al. 2008; Day et al. 2015). As extreme examples, consider the wide/flat skulls and robust hyoids of suction-hunting catfishes and aquatic amphibians (Van Wassenbergh et al. 2006; Carreño and Nishikawa 2010; Heiss et al. 2013; Wainwright et al. 2015). Data on foraging behavior in cave-adapted *A. mexicanus* are abundant (Kowalko et al. 2013; Espinasa et al. 2014; Kasumyan and Marusov 2015; Espinasa et al. 2017). Nevertheless, there has been little to no focus on foraging kinematics beyond divergence in dentition and jaw dimensions (reviewed by Jeffery 2020). We suggest a more thorough examination and comparison between cave and surface functional morphology and feeding kinematics in *A. mexicanus* would be fruitful.

In addition, we noted that cavefish jaws were slightly agape in their resting state, relative to surface fish. This occurred across stages and populations, and does not appear to be due to fixation artifact. It is possible that this feature is associated with the divergent size and/or positioning of the cavefish hyoid, which is physically linked to the mandible via the geniohyoideus muscle and drives lower jaw opening via hyoid depression (Lauder 1981b; Hernández et al. 2002); however, there are many other soft-tissue attachments onto the mandible, underscoring the need for functional/kinematic analyses of feeding in this system. It is also possible that mouth positioning represents an adaptation associated with enhanced sensory perception. In the study by Holzman et al. (2014), the authors found that when introduced to a novel environment cavefish rapidly opened and closed their mouths, using suction to generate a velocity field around their heads. They posit that the mechanism is analogous to echolocation and can be used for object detection by neuromasts, which are expanded in cavefish versus surface fish. We note that in the article's figures and highspeed movies the mouths of representative cavefish are never fully closed, which is consistent with our findings and laboratory observations. Given that tastebud expansion represents another cave-adapted trait in *A. mexicanus*, it is possible that an open mouth posture has evolved to facilitate fluid flow over tastebuds for enhance chemoreception. Fish use their sense of taste not only to find and select food, but also to sense their environment in a more general way (reviewed by Kasumyan 2019). While any adaptive explanation for this (generally subtle) phenotype remains speculative, we suggest that it merits further inquiry.

### Conclusions and looking forward

Owing to their dramatic, extensive, and repeated adaptation to caves, *A. mexicanus* is emerging as a popular model system to examine the development and evolution of an array of traits, including but not limited to anatomy, behavior, immunity, regeneration, and metabolism (Ponnimbaduge Perera et al. 2023). Further, with the rapid development of genetic/genomic tools and resources (Ponnimbaduge Perera et al. 2023), these fish are now being used in research universities and medical schools worldwide. A compelling attribute of this system is the widespread convergence of cave-adapted traits across populations, allowing biologists to assess the extent to which genetic convergence underlies convergence in phenotype (e.g., Riddle et al. 2018). Our results suggest that global aspects of craniofacial geometry are nonconvergent and that population demographics—not developmental constraints—shape levels and patterns of variation within and across populations. However, some commonalities we noted for cave populations, including wide jaws, shallow sloping skulls, and shifted hyoids, which all suggest adaptations toward the generation of suction, whether for feeding or object finding. Combining genetic and kinematic tools in this system holds much promise for expanding the evo-devo paradigm (Irschick et al. 2013).

In addition, we note that craniofacial differences arise early in development, likely before 4 days, in *A. mexicanus*. This observation is in line with other characterized surface: cave differences in gastrulation, oocyte provisioning, dental patterning, and neural crest cell development (Atukorala and Franz-Odendaal 2018; Yoshizawa et al. 2018; Torres-Paz et al. 2019; Riddle et al. 2020; Gross et al. 2023), and suggests that evolved differences may arise during early embryonic patterning events. In a similar study, Power and colleagues (2017) documented subtle differences in chondrocranial shape between juvenile (6–8 dpf) surface fish and cavefish. Given the ability to manipulate the developmental genetic toolkit in *A. mexicanus*, identifying the proximate molecular mechanisms that underlie divergence in craniofacial form appears tractable in this system. We note further that shape differences between cave populations are as pronounced as between cave and surface populations, especially at earlier stages (e.g., some of the largest pairwise effect sizes were for Molino:Tinaja). While population genetic/genomic studies focusing on *Astyanax* routinely incorporate multiple cave populations (Bradic et al. 2012; Gross 2012; Bradic et al. 2013; Herman et al. 2018; Ponnimbaduge Perera et al. 2023), the literature on phenotypic variation explicitly examines differences among cave populations to a lesser extent (but see Powers et al. 2017). We feel that this area is worthy of additional investigation, especially for functionally relevant aspects of craniofacial shape.
